# Effect of comprehensive psychosomatic promotion in hypertension patients with anxiety and depression based on community: A randomized parallel controlled trial

**DOI:** 10.1097/MD.0000000000021451

**Published:** 2020-08-14

**Authors:** Hailiang Zhang, Xiaomei Jiang, Haixia Da, Runjing Dai, Na Zhao, Weimin Pan, Jingchun Fan

**Affiliations:** a School of Public Health, Center for Evidence-Based Medicine, Gansu University of Chinese Medicine; b Psychosomatic and Sleep Medicine, Gansu Gem Flower Hospital; c Department of Mental Health, Gansu Provincial Centre for Disease Control and Prevention, Lanzhou City, Gansu Province, P.R. China.

**Keywords:** anxiety, depression, hypertension, mindfulness, psychotherapy

## Abstract

**Background:**

Mental health is closely related to the occurrence of hypertension, particularly the prognosis of hypertension patients. The role of psychotherapy in the occurrence, development, prevention, and prognosis of hypertension, remains to be clarified.

**Methods/design:**

We will conduct a prospective, double-blind, randomized, multiple-centers study. Eighty patients enrolled in this trial will be randomized at 1:1 ratio. The primary endpoint is will be the reduction of the patient psychological scale (PHQ-9) score. Secondary endpoints will be the drop in blood pressure, awareness of physical and mental health and self-efficacy scale. Measurements will be performed at baseline, 5-week (questionnaires only), 10-week (primary endpoint), using the Anxiety Screening Questionnaire (GAD-7) and Depression Scale (PHQ-9). Data analysis will be carried out using the SPSS v.25 software assuming a level of significance of 5%. Results will be analyzed using multilevel, regression analysis and hierarchical linear models.

**Discussion:**

We hope to provide some insight in the understanding the underlying mechanism of the novel mindfulness in the management of hypertension related psychological stress/disturbance, and will enable us to develop novel approach to manage essential hypertension and its related psychological disorders.

**Clinical trial registry:**

http://www.chictr.org.cn (ChiCTR1900028258)

## 1 Introduction

With the change of the “biological-psychological-social” medical model, it is agreed that essential hypertension (EH) is a typical psychosomatic disease. EH accounts for about 90% to 95% of hypertension patients. The prevalence of hypertension in adults (18 years) is 27.9% in China.^[1]^ The number of hypertension patients is exceeded 270 million. The prevalence of hypertension is doomed to increase in the next few decades, due to increase longevity and lifestyle changes.^[2]^ In addition to the body itself or genetic factors, the psychological factors contribute to occurrence of hypertension. Hypertension caused by long-term depression and anxiety, compromises the quality of life and poor prognosis.^[3]^ It has been demonstrated that the patients with cardiovascular disease have a higher incidence of psychological disorders, as well as increased risk of central cerebrovascular accidents in people with anxiety and depression.^[4]^ Psychological stress increases the risk of developing high blood pressure, which also positively contributes to the high incidence of psychological and behavioral disorders.^[5]^ Some overseas studies demonstrate that the prevalence of depression, anxiety disorder or the mixed is 10.3%, 27.1%, or 4.5%, respectively.^[6]^ On the other hand, The Chinese Mental Health Survey shows that the prevalence of anxiety or mood disorders is 7.57%, or 7.37%, respectively.^[7]^ Thus it is logical to understand that the hypertension patients are vulnerable with mental health problems.^[8]^

Hypertension, a lifelong disease, is often accompanied with left ventricular hypertrophy, coronary heart disease and stroke and kidney failure. The management of hypertension is rather lengthy challenge, often requires patient life-time self-management.^[9]^ Because hypertension is a life-time disease, these patients may lead to ideological burden and psychological stress.^[10]^ It is well known that hypertension patients are more likely to have negative emotions such as fear, anxiety, and depression.^[11]^ However hypertensive patients with psychological problems (such as depression or anxiety) are often not to stick to medication, leading to unstable blood pressure, more prone to complications, and finally lowering the quality of life.

Hypertensive patients often experience negative emotions with anxiety and depression during treatment and are often in a state of survival, further exacerbating their negative emotions.^[12]^ Thus, to solve these negative emotions is a key in improving the quality of life. Mindfulness-based cognitive therapy is mainly use to explore the effectiveness of treatment of hypertension with anxiety and depression,^[13–15]^ reducing stress through mindfulness, and adjusting the psychology to achieve the effect of reducing blood pressure.^[16,17]^ Cognitive behavioral therapy (CBT) is widely used in treatment of hypertension patients, because of its advantages of wide benefit and high efficiency. CBT provides face-to-face communication opportunities and, makes the patients familiar with the environment quickly to increases the sense of security and reduces their discomfort. Although the research on mindfulness based stress reduction is not rich, it describes an alternative approach for physicians to help these patients cope with their condition more effectively.

The research of CBT for hypertension patients in China is at the initial stage. The problems include a relatively small sample, non-standard mindfulness interventions, and a high rate of loss. In addition, the researchers only focus on Explain whether hypertension is effective without quantitation of hypertension value, nor epidemiology. Thus ideally the research should be focusing on the mechanism of mindfulness therapy in the anti-hypertension with objective quantification analysis.

## 2 Methods/design

### 2.1 Trial design

**Figure 1:**
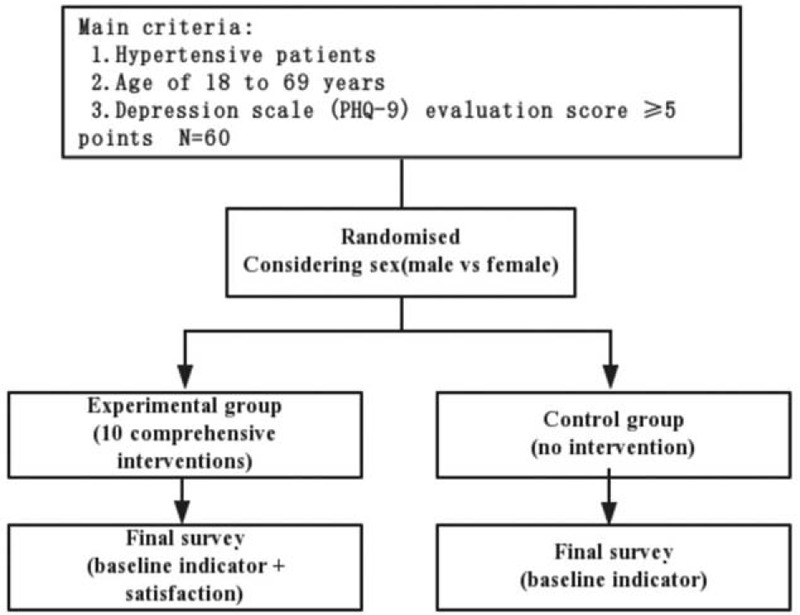
Protocol scheme of the study.

**Figure 2:**
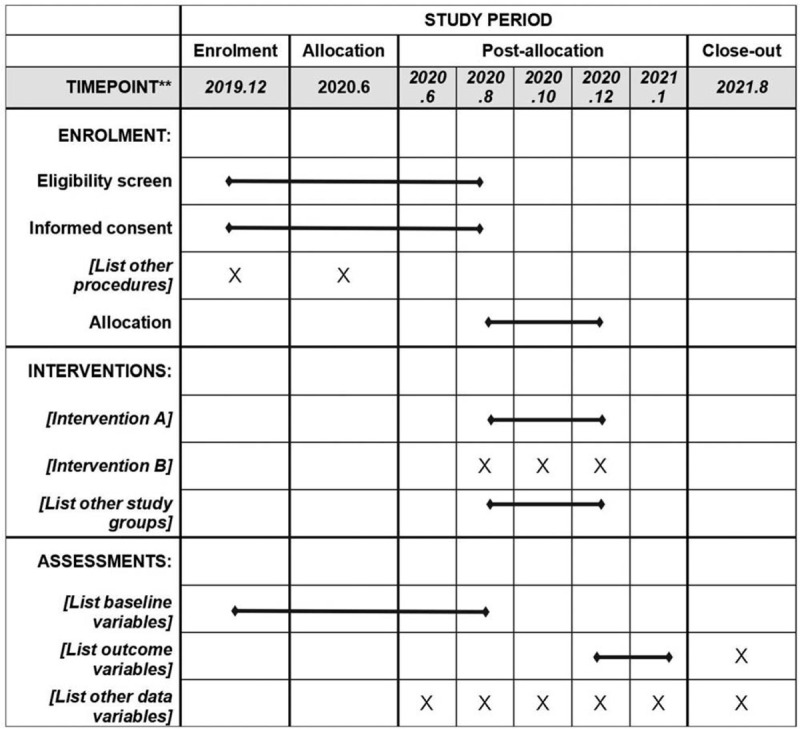
The schedule of enrolment, interventions, and assessments.

This study is designed as a prospective, double-blind, randomized, multiple-center study with 10 weeks duration. The protocol scheme (Figs. 1 and 2).

### 2.2 Aim of the study

The objective of this study is to perform comprehensive psychosomatic promotion in hypertension patients with anxiety and depression for improvement of their physical and mental health status. Furthermore we also promote the awareness of physical and mental health knowledge, particularly in self-management.

### 2.3 Eligibility criteria of the participants

#### 2.3.1 Inclusion criteria

1. Hypertensive patients (systolic blood pressure > 140 mmHg, and/or diastolic blood pressure > 90 mmHg)
2. Depression scale (PHQ-9) evaluation score ≥ 5 points
3. Aged 18 to 69 years
4. Understand the questionnaires without obvious barriers to daily communication
5. Stay in the current residence within the next 6 months.

#### 2.3.2 Exclusion criteria

1. Patients are with severe myocardial infarction, cerebral infarction, cirrhosis, uremia
2. Physically unable to participate in the project activities
3. Vision and hearing were severely impaired, and physical activity is limited affecting participants
4. Taking anti-psychotics, anti-anxiety and depression medications within 3 months
5. Taking self-reported with substance abuse and Alzheimer's disease.

### 2.4 Discontinuation criteria

1. Disease progression or death.
2. Patient refusal.
3. Other reasons of unsuitability owing to which the study protocol should not continue.

### 2.5 Enrollment, randomization and blinding

We plan to recruit 80 cases and actually 60 people; patients will be randomized at 1:1 ratio in the Gansu Gem Flower Hospital. The study participants do not know their grouping, the outcome assessors and the analyst do not know the grouping of the research objects, are responsible for collecting the baseline data, the physical examination, questionnaire survey at the end of the study, and conducted data entry and statistical analysis. The blinding codes will place in a sealed envelope and will be revealed only when adverse events occur or at the terminal analysis.

### 2.6 Background data

The patients’ background data are collected prospectively, including sex, age, history, complications, past treatments for hypertension. The results of the project are only published in groups, and no personal information will be published; the information of the questionnaire you fill out will be kept in a safe and reliable place and will not be disclosed to members outside the research team. We will strictly protect your personal information. The data was obtained by contacting the corresponding author 6 months after the test results were published. Monitoring will be performed annually by each dataset to evaluate and improve study progress and quality.

### 2.7 Statistical analysis

The primary population for efficacy analysis is the intention-to-treat population, defined as all randomized patients. The stratified OR will be estimated by logistic regression analysis with randomized factors as covariates, and multivariate regression analysis will be used to explore interactions between therapies and back-ground factors. Linear models will be used to test for between group differences in the continuous outcome measures. Secondary endpoints are summarized using 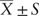 and frequency. Missing data will be excluded from the analysis of the corresponding endpoint.

### 2.8 Interventions

The comprehensive intervention measures of the trial group will be the teaching and practice of psychological comfort adjustment technology, whereas the control group will attend health education talks. In this study, blind method will be applied to the research objects, double blind fashion. All participants receive ten ninety-minute treatments over 10 weeks and once a week, divided into physical health and psychological section. Treatments are provided at no cost to participants. The contents of 10 activities in the intervention group and the control group are shown in Table 1.

**Table 1:**
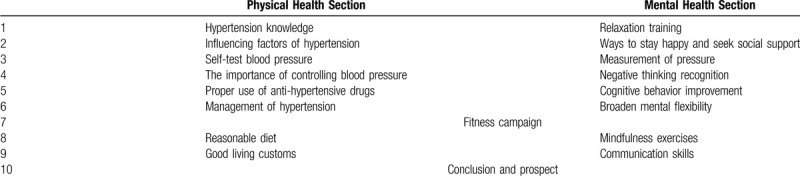
Details of each regimen.

### 2.9 Outcomes

Primary endpoint is the psychological scale score. Secondary endpoints are blood pressure in these patients, awareness of physical and mental health, and self-management efficiency. We will measure these parameters separately in three time-points, that is, base, mid court (the fifth week), and final lines. We will evaluate the psychological scale score, blood pressure, physical and mental health awareness and self-efficacy at week 10.

### 2.10 Sample size calculation

The sample size will be calculated by PASS software based on the difference test formula for 2 samples mean comparison in group randomized controlled trial. The indicators will be selected as follows:

(1) The error setting is 1−*β *=* *0.9, *α *=* *0.05.
(2) Sample size setting: There are two groups for each community in the intervention group and the control group, with about 30 people in each group. The number of communities in the intervention group and the control group is same.
(3) Effect scale setting: there is no change in the control group after the intervention, and the mean change is calculated based on the anxiety end-line and baseline difference of the intervention group in the pretest hypertension patients. Intragroup correlation was set to 0.01.

### 2.11 Informed consent

Signed informed consent will be obtained from all of the enrolled patients. Any of these participants can withdraw from the study without restrictions. The data collected up to the time of withdrawal will be included in the analysis unless participants specifically request for their data to be withdrawn.

### 2.12 Ethics approval and dissemination

This study has been approved by the Human Ethics Committee, Gansu Gem Flower Hospital (approval number: 201902), *informed consent to participate will be obtained from all participants*. The results of this study will be published in a peer-reviewed journal.

### 2.13 Patient and Public Involvement

Neither patients nor the public were involved in the conception or conduct of the study.

### 2.14 Limitations of this study

Intrinsic and extrinsic factors influencing physical and mental health factors are diverse, therefore, the relative importance of circumstances may vary with time and this needs to be considered when designing the programs and policy interventions.

The current study will be performed in 3 areas of Lanzhou, which doesn’t necessarily reflect the entire country, the possible treatment bias may not be excluded completely.

Moreover, we realize that the intervention implementation process, team training, different times, resources and adaptations, may have some impacts to the outcomes of the study.

## 3 Discussion

Mindfulness, an emerging branch of psychotherapy, has a unique role in the treatment of hypertension with anxiety and depression. However, the group interventions have not been established to promote the positive development of physical and mental health in hypertension patients in the community.

Therefore, we have selected 3 areas in Lanzhou to confirm the hypothesis mentioned above, and hopefully to provide some insight in the understanding the underlying mechanism of the novel mindfulness in the management of hypertension related psychological stress/disturbance. In addition, we aim to use this protocol to investigate the potential role mindfulness in management of other chronic devastating conditions in the body, in addition to chronic hypertension in the future.

Finally, this protocol would enable us to develop novel approach to manage essential hypertension and its related psychological disorders. We understand there are some limitations in the current protocol, which will be improved while we start our study in future.

## Acknowledgments

The authors thank Caiqin Xi, Yinping Liu and the staff based at Psychosomatic and Sleep Medicine, Gansu Gem Flower Hospital for their hard work and assistance with recruitment and survey management, and deep gratitude to the patients involved in our trial.

## Author contributions

HLZ, XMJ, HXD, RJD, NZ, WMP, and JCF all contributed to the development of the study protocol. HLZ and XMJ wrote the manuscript. JCF and WMP conceived the trail and provided methodological advice, polished and revised the manuscript. HXD, RJD and NZ enrolled participants collected the data and assign participants to interventions. JCF will be the third senior reviewer that will help resolve any discrepancy and approved the final version of the manuscript. All authors approved the final version of the manuscript.
